# The Evolution of Neuromodulation in the Treatment of Chronic Pain: Forward-Looking Perspectives

**DOI:** 10.1093/pm/pnz074

**Published:** 2019-06-01

**Authors:** Michael A Fishman, Ajay Antony, Michael Esposito, Timothy Deer, Robert Levy

**Affiliations:** 1 Center for Interventional Pain and Spine, Exton, Pennsylvania; 2 University of Florida, Gainesville, Florida; 3 Florida Pain Institute, Melbourne, Florida; 4 The Spine and Nerve Center of the Virginias, Charleston, West Virginia; 5 Institute for Neuromodulation, Boca Raton, Florida, USA

**Keywords:** Technology, Stimulation Patterns, Closed-Loop, Miniaturization, Noninvasive, Optogenetics

## Abstract

**Background:**

The field of neuromodulation is continually evolving, with the past decade showing significant advancement in the therapeutic efficacy of neuromodulation procedures. The continued evolution of neuromodulation technology brings with it the promise of addressing the needs of both patients and physicians, as current technology improves and clinical applications expand.

**Design:**

This review highlights the current state of the art of neuromodulation for treating chronic pain, describes key areas of development including stimulation patterns and neural targets, expanding indications and applications, feedback-controlled systems, noninvasive approaches, and biomarkers for neuromodulation and technology miniaturization.

**Results and Conclusions:**

The field of neuromodulation is undergoing a renaissance of technology development with potential for profoundly improving the care of chronic pain patients. New and emerging targets like the dorsal root ganglion, as well as high-frequency and patterned stimulation methodologies such as burst stimulation, are paving the way for better clinical outcomes. As we look forward to the future, neural sensing, novel target-specific stimulation patterns, and approaches combining neuromodulation therapies are likely to significantly impact how neuromodulation is used. Moreover, select biomarkers may influence and guide the use of neuromodulation and help objectively demonstrate efficacy and outcomes.

## Eras in Spinal Cord Stimulation

The year 2017 marked the 50th anniversary since the first reported use of dorsal column electrical stimulation to treat pain [1,2]. Originally based on the proposed concept of the gate control theory of pain [3], spinal cord stimulation has advanced quite rapidly in recent years [4–7]. Mechanisms of action beyond gate control theory, such as the direct modulation of wide–dynamic range neurons, the modulation of activity in cortical and subcortical brain regions, and activation of descending inhibitory pathways, have been proposed [8].

The first spinal cord stimulator (SCS) was made commercially available in 1968. This technology used a radiofrequency receiver coupled with dorsal column–stimulating electrodes and an external power supply [9]. Surgical complications and practical considerations gave way to the first fully implantable spinal cord stimulation system in 1981 [10]. The first rechargeable implantable pulse generator (IPG) was introduced to the market in 2004 [11]. Innovation in the field after Food and Drug Administration commercial approval focused on decreasing the size of the IPG and improving the stability and maneuverability of leads [12,13]. The evolution of leads, including multicolumn and multicontact leads, led to a significant decrease in surgical revision rates [14].

An initial trend showed a tendency to favor rechargeable systems due to smaller IPG size and longer lifespan [15]. The rationale behind this was mainly focused on cost-effectiveness and decreased potential for complications, with fewer IPG replacements needed over time [16]. However, recent studies have consistently shown higher rates of explanation for a rechargeable system when compared with a primary cell IPG [17,18]. Also, it is not entirely clear that smaller rechargeable devices are what patients prefer, as opposed to having to endure the burden of recharging the device, although there may be a size-to-charging burden ratio that would favor a smaller profile [19]. Research on efficacy, at times lacking both in quantity and quality, has historically focused on a limited number of clinical applications such as postlaminectomy syndrome and complex regional pain syndrome [20,21]. In the past several years, high-quality level 1 evidence has been generated in the field of neuromodulation for pain treatment [4,5,7]. With emerging technology involving novel targets and pulse trains, a trend toward paresthesia-free stimulation modalities has been observed. In its present form, neuromodulation is used as a generally safe and reversible therapy that is used to treat chronic pain, decrease the need for systemic medications, including opioids, and more efficiently utilize health care resources [13,22,23].

## Device Programming and Electrical Fields

Traditional SCS programming has involved selecting various combinations of lead cathodes (-) and anodes (+) in order to shape an electrical field within the dorsal columns with the goal of creating overlapping paresthesias in painful areas. Manipulation of stimulation parameters (apart from contact selection), including frequency, amplitude, and pulse width, can impact the strength, intensity, and perception of associated paresthesias from the device. Creative arrays or configurations make it possible to preferentially target specific fibers within the dorsal column [24]. However, conventional tonic paresthesia-based stimulation has limitations. Postural changes affecting the distance of the spinal cord from the leads can result in changes in the perceived strength of the stimulation [25]. Additionally, paresthesias may be felt outside of the painful region and can be uncomfortable for the patient [26]. For example, a patient requiring paresthesia coverage solely for low back pain may experience intense paresthesia in the legs in an effort to capture a therapeutic effect on the low back. Moreover, given the anatomical distribution of the axons in the dorsal columns at the midthoracic area, it can be challenging to effectively modulate fiber physiology innervating various axial structures with lower tonic stimulation frequencies [27]; it should be acknowledged, however, that recent improvements in technology make capturing the low back much more feasible than in past decades. Although SCS has been effectively utilized with traditional paresthesia-based programming, it is clearly not without limitations. Despite these shortcomings, traditional stimulation has yielded relief for many patients with moderate efficacy; even given the demonstrated effectiveness of some novel pulse trains, some patients prefer paresthetic tonic stimulation [28,29].

## A New Era—SCS Patterns

With the increasing study of spinal cord stimulation physiology, the concept of altering stimulation parameters to subsequently modulate the pathophysiology in chronic pain has become the focus of therapy development [8,30,31]. For decades, it has been known that the nervous system uses “codes” to encapsulate and transmit information. Patterns of stimulation can evoke very different responses in multiple regions of the central nervous system, and thus provide an opportunity to better address the complex neurophysiology in chronic pain. Moreover, clinical research on the contemporary use of newer stimulation patterns has focused on generating high-level evidence to support therapeutic outcomes.

### kHz Frequency SCS

In contrast to traditional tonic stimulation, which typically is programmed between 40 and 100 Hz, higher frequencies of SCS (HF-SCS) have also been tested up to 10 kHz [7,32,33]. One of the results of HF-SCS stimulation is the absence of sustained action potential generation in the dorsal columns, despite current amplitudes being at or above threshold [34]. This, combined with lower thresholds and pulse amplitudes, effectively reduces the likelihood of a patient feeling paresthesia [31]. In contrast to traditional SCS lead placement, which is based on intraoperative paresthesia mapping, leads for HF-SCS are anatomically placed to span the T9-10 interspace [35]. Multiple published studies, including prospective, open-label studies and RCTs, have shown that HF stimulation can provide significant pain relief for the treatment of back and leg pain [7,32,33,35,36]. The SENZA RCT study, utilizing a stimulation frequency of 10 kHz, found superior pain relief when compared with tonic, low-frequency (LF) stimulation. The control group utilizing tonic-LF stimulation demonstrated typical outcomes for this therapy modality in low back pain patients with a component of leg pain. Patient-reported pain relief in this study was maintained out to two years [37].

The relation between stimulation frequency and patient-reported outcomes was recently studied by Al-Kaisy and colleagues [32]. This sham-controlled RCT found that higher frequencies (5,882 kHz) showed a larger decrease in patient-reported pain when compared with lower kHz frequencies (1,200 and 3,030 Hz). Interestingly, the sham control group demonstrated comparable relief to the lower kHz HF-SCS groups. Another sham-controlled study using 5,000-kHz stimulation in tonic-LF responders showed no difference vs sham in responder rate using the Patient Global Impression of Change and visual analog scale (VAS) score reductions [33].

As with all sham-controlled trials in neuromodulation, there are inherent difficulties in running these types of studies. They often lead to more questions about results and study designs, as well as the utility of the treatment under investigation. In 2013, a European study reported significant reductions in VAS using HF-SCS in 74% of patients at six months [35]. Al-Kaisy et al. reported results from 198 patients with chronic intractable back and leg pain enrolled in a prospective, randomized study, comparing HF SCS with tonic stimulation. Patients experienced significant reductions in VAS for not only leg pain but also traditionally difficult-to-treat back pain that were durable out to 24 months [36]. Reductions in scores (indicating improvement) on the Oswestry Disability Index (ODI) and reduced opioid usage were also reported. Investigators reported a responder rate of >80% for both back and leg pain in the HF-SCS group, compared with responder rates in the traditional SCS group of 43.8% and 55.5% for back and leg pain, respectively. The superiority of 10-kHz stimulation over traditional SCS was seen through 12 months [7].

HF stimulation has also been reported to improve axial low back pain in patients without prior back surgery [38]. In contrast to these results, two other published studies, including an RCT, did not support these results [39,40]. Both the Russo et al. and De Andres et al. studies found lower effectiveness (<50% pain relief) in the HF-SCS groups when compared with the aforementioned pivotal RCT. Recently, Thomson and colleagues published results from the PROCO study [41], demonstrating equivalent pain relief with SCS at 1-, 4-, 7-, and 10-kHz SCS. These latter findings suggest that lower frequencies within the kHz range might be able to produce similar clinical results at 10 kHz with considerable energy savings.

The mechanisms of action of HF-SCS have not been clearly defined. Several “working hypotheses” have been proposed via animal and computational models that include wide–dynamic range (WDR) neuron modulation, dorsal horn fiber recruitment, and local depolarization blockade [34,42,43]. However, some modeling studies have suggested that local blockade does not occur at clinical amplitudes [44]. Human studies have also shown a lack of efferent motor activity with kilohertz frequency SCS, consistent with the abbreviated DC fiber activation patterns seen in animals [34,45]. Thus, although the clinical findings are promising, the underlying mechanism(s) of action using 10-kHz stimulation remain to be elucidated. A unique observational case series examining intraoperative electromyography (EMG) with HF-SCS stimulation showed no observable EMG responses with 10,000-Hz stimulation. This contrasted with other observed waveforms, including traditional tonic and burst SCS, which displayed various signal-generating thresholds, distal to proximal muscle activation, and a phenomenon of hyperexcitability, suggesting different underlying mechanisms of action [45].

### Burst SCS

Burst is another novel stimulation pattern that utilizes a specific pulse train [46–48]. De Ridder et al. first described burst stimulation (five 1-ms pulses with a 1-ms interpulse interval at 500 Hz, applied at a 40-Hz frequency with passive regeneration of the charge balance at the end of the pulse burst) using cortical stimulation for the treatment of tinnitus [49,50]. Neuronal bursting is a well-studied phenomenon that helps code presynaptic information and responses that have a multitude of postsynaptic effects including temporal summation, short- and long-term potentiation, and information filtering. Classically defined burst effects have been described from the spinal cord up to thalamocortical relay centers [51–53]. Multiple elements of the stimulation pattern are central components of the burst signal. These include basic parameters such as pulse width and amplitude, but also aspects of the pulse burst itself such as the interburst timing and burst signature. All of these components affect the dimension and dynamics of the electrical field and impact the function of the target tissues being directly influenced by the field. Moreover, these effects will also indirectly impact structures downstream from the targeted neural tissue. The importance of the specific burst signature and parameters has been studied using computational models, as well as in animals and humans [30,45,54,55]. What may seem like subtle differences in signature (e.g., passive vs active recharge) can have a cascading effect on primary and secondary neural elements. Moreover, specific timing elements are also important, which is not surprising as neuronal channel kinetics and synapses are highly sensitive to spike timing. Thus, slight differences in evoked ensemble responses in the DC from variations in a burst pattern can have meaningful neurophysiologic ramifications.

Burst SCS has been clinically evaluated in multiple prospective studies, including sham-controlled and comparative RCTs [4,56–60]. The SUNBURST study demonstrated the statistical superiority of pain ratings with burst stimulation relative to pain ratings with tonic stimulation, and the majority of study subjects preferred burst stimulation over tonic due to better pain relief and lack of paresthesias [4]. Clinically, burst stimulation has been studied in the treatment of diabetic neuropathy, complex regional pain syndrome, and failed back surgery syndrome, with the predominant research being conducted on low back and leg pain. Burst SCS has also been shown to recover analgesia in patients who have failed, or shown accommodation to, tonic SCS [61]. Both electroencephalography (EEG) and positron emission tomography–computed tomography imaging studies have shown that burst SCS demonstrates differences in cortical and subcortical cerebral activation patterns, suggesting that this waveform may have the ability to directly and differentially modulate the brain regions involved in the emotional and affective aspects of the pain experience [46,62]. Mechanistically, burst SCS has been shown to involve different neurochemical pathways in the dorsal horn of the spinal cord and also have a pronounced effect on WDR neurons within the spinothalamic tract [63,64]. Similarly, burst SCS does not seem to alter firing rates of neurons in the dorsal column nuclei, suggesting that there is an enhanced ability to modulate pain processing in the dorsal horn without activating the neuronal fibers, which would create the sensation of paresthesia [64]. This difference in the ability to selectively modulate different somatic nerve processing is a possible mechanism for the resulting paresthesia-free analgesia.

### High–Pulse Width SCS Programming

Another stimulation method that is being explored comprises a higher-charge delivery (or density) over a given period of time. Typically, this approach has been programmed at subparesthetic levels. Increasing pulse width and frequency increases the charge delivered per second [65]. It is hypothesized to provide an increased ability to modulate neural structures without activating fibers to the extent that it generates paresthesias. A critical element of this paradigm would be the frequency component; otherwise the increased pulse width would simply follow a strength–duration curve [66,67]. Provenzano et al. reported a retrospective review on patients using high-density stimulation that showed no significant difference when compared with conventional paresthesia-based stimulation, thus bringing into question the clinical utility of the technique [68]. However, alterations in programmed electrical parameters continue to be an area of exploration [69].

### Random/Stochastic Stimulation Patterns

Several disorders of the central nervous system (CNS) are characterized by abnormal neuronal synchrony. Random stimulation was developed to selectively counteract abnormal neuronal synchrony by eliciting desynchronization [70–73]. Random stimulation patterns could evoke antikindling or correct alterations of abnormal synaptic connectivity and synchrony. Zeitler et al. demonstrated that a random slowly varying sequence (SVS) stimulation pattern significantly improved the antikindling effect of random stimulation and tremor suppression [74]. Further support for this concept has been published by Manos et al., demonstrating that there is an optimal frequency and intensity of random acoustic patterns in the treatment of tinnitus. These findings suggest that random stimulation pattern usage may be co-dependent upon other stimulation parameters [75]. Adamchic et al. also demonstrated that the type of stimulation is important for acute and long-term effects in acoustic neuromodulation for durable improvement in tinnitus symptomatology [76]. Patient-reported tinnitus improvement was correlated with EEG recordings, demonstrating an objective, electrophysiologic biomarker for improvement. The application of random patterns is not limited to the auditory cortex, but also subcortical networks involved in movement such as the basal ganglia [70,77].

Stochastic approaches have also been suggested for peripheral afferent sensory fibers in the treatment of tremor [78]. In a slightly different paradigm, Dideriksen et al. demonstrated the suppression of pathologic tremor through electrical stimulation of afferent fibers. In this case, either surface or intramuscular EMG recordings were used to detect tremor and time stimulation. Different stimulation timing (in-phase or out-of-phase with tremor) and parameters were trialed, and the data suggest the need for patient-specific stimulation protocols for tremor suppression [79]. This concept is not foreign to neuromodulation for the treatment of pain, as physicians must personalize treatment by choosing the right device and stimulation settings for each patient. Although randomized stimulation patterns have shown promise for longer residual effects on neurologic function [77], it is unknown how different systems and underlying conditions may respond to this type of stimulation.

## Therapy Tolerance and Habituation

Loss of therapeutic effect is the most common reason for SCS therapy failure over time [80]. This has been demonstrated in both the United States and Europe through the examination of explant data [17,18,81]. Although the loss of efficacy could be due to a variety of reasons, habituation or tolerance to neurostimulation has been one of the greatest challenges to long-term efficacy in neuromodulation for chronic pain [26]. As new technologies are being developed to improve efficacy and therapy response rates, physiologic adaptation to therapeutic stimulation remains a challenge for the entire field. Future devices may utilize multiple stimulation strategies or a more “random” pattern of stimulation (see *Random/StochasticStimulation Patterns*) in order to help mitigate CNS adaptation over time [82]. Other sources of inadequate analgesia include disease alteration and progression and alterations in patient psychological status. Habituation of neurostimulation for chronic pain is poorly understood, but there are common physiologic examples of the human body adapting to a new “set point” in a disease-based maladaptive homeostatic change. In hypertension, cerebral autonomic regulation adjusts a set point over time as part of the pathophysiologic state [83]. Of course, tolerance and adaptations to centrally acting pharmaceuticals are also well-studied phenomena, with a myriad of underlying mechanisms, including but not limited to neurochemical receptor endocytosis, activation of intracellular second messenger systems, and transcriptional control of cellular function [84]. It is reasonable to assume that a similar principle may govern the body’s adaptation to neurostimulation over time. In fact, as an external stimulus acting through multiple electrophysiological and neurochemical mechanisms, it would be surprising to not see some form of adaptation with neural stimulation. However, because different stimulation patterns and signatures likely evoke different cellular mechanisms of analgesia, it is possible that patients who fail one form of stimulation may achieve analgesia with another. This phenomenon is supported in clinical reports of patients who have failed tonic stimulation and subsequently experienced improvements when switched to burst SCS [61,85]. We have yet to see how newer waveforms and pulse trains will perform over time as a salvage solution to short- and long-term SCS failures. Future research is warranted to investigate this possibility.

## Feedback Control of Neurostimulation

Virtually all neural systems in the body utilize feedback from the environment or the body habitus to maintain homeostasis. Feedback in the skeletal motor, autonomic, and sensory systems provides a much finer fidelity and accuracy of controlling physiologic function. A feedback-based system, or closed-loop control, is based upon the classic homeostatic mechanism whereby a biosignal is detected and measured, and the resulting signal is then used to adjust physiologic function. For example, blood pressure is partly controlled via baroreceptors in the carotid sinus. The baroreceptors detect a change in pressure, then rapidly adjust arterial pressure through autonomic control of heart rate, stroke volume, and total peripheral resistance. There are multiple examples of medical device-based approaches that utilize biomarker feedback, including deep brain stimulation for Parkinson’s disease [86–88] and epilepsy [89,90], cochlear stimulation for hearing, and spinal cord stimulation for chronic pain [6,91,92].

Conventional neuromodulation is based on an open-loop paradigm, in which stimulation parameters are statically set by a clinician and changes in stimulation intensity (pulse amplitude) are made by the patient. This strategy poses several challenges with SCS, especially as the most common anatomic location for stimulation is the midthoracic spine, corresponding to back and leg pain targets [93]. There are a number reasons why conventional SCS has traditionally had limited success in treating axial back pain. The low back is not particularly well represented in the spinal cord as compared with other areas of the body, such as the hands or feet, because the back is not used for fine touch or sensation. There are comparatively fewer sensory fibers corresponding to the low back, and these fibers tend to be deeper and also more lateral in the spinal cord [93]. The dorsal cerebrospinal fluid (dCSF) layer is thickest around the midthoracic spine, such that stimulation in this area requires more energy to cross that fluid barrier; in fact, only 10–20% of the current from spinal cord–stimulating electrodes provides stimulation of the dorsal columns. To complicate matters further, the dCSF thickness changes positionally (i.e., thicker when patients are prone and thinner when patients are supine) [94]. This creates a clinical and technical dilemma—different amounts of energy are needed to pass current across the fluid layer and can lead to supratherapeutic or subtherapeutic stimulation. There are a number of physiologic parameters that further influence the energy requirements and SCS experience, including dynamic factors such as heartbeat and respiration that continuously vary. On one hand, this can lead to unwanted or unpleasant stimulation, and on the other hand, it may lead to understimulation and lack of efficacy.

The first commercially available closed-loop SCS system involved detection of body position, rather than a neural signal [92]. This system used a gravitational signal to help control intensity of neurostimulation, which fluctuates with bodily position (supine vs upright). The system uses a three-axis accelerometer, which is calibrated at a baseline visit to recognize different body positions and programmed with patient-specific stimulation parameters for each position to avoid over- or understimulation. A prospective, multicenter, open-label randomized crossover study of 79 patients comparing automatic position-adaptive stimulation with conventional manual programming found a 41% reduction in number of daily patient-directed programming button pushes (18.2 vs 30.7 per day) [92]. Not surprisingly, using body position as a feedback loop for neurophysiologic parameters does not account for all variables involved in maintaining stimulation in the therapeutic window but served as an incremental improvement over conventional stimulation.

Toward developing a feedback-controlled system in SCS, Parker et al. reported measurements of evoked compound action potentials (ECAPs) from the spinal cords of sheep as well as patients undergoing stimulation for pain relief [91,95]. This represents an objective electrophysiologic biomarker in response to neurostimulation of the dorsal columns. Furthermore, it has been demonstrated that the amplitude of sheep A-beta fiber potentials during SCS exhibits dependence on electrode location, highlighting the potential for optimization of A-beta recruitment, pain relief, and power consumption in SCS devices [95]. From these observations, the idea of a closed-loop system with feedback to adjust “dose” of stimulation input to achieve constant neural recruitment and to avoid both overstimulation and inadequate pain relief was initially tested. In a recently published study, Russo and colleagues demonstrated the clinical effectiveness of a closed-loop SCS system utilizing low-frequency tonic stimulation and an improvement in neuromodulating a well-established target, the dorsal columns [6]. Pain was significantly reduced in the legs and, to a greater degree, in the back. This suggests that there is good recruitment of back-specific dorsal column fibers, although the exact mechanisms are yet to be elucidated. Furthermore, a significant advantage of a closed-loop feedback-based SCS system may be its prevention or limitation of stimulation amplitudes reaching the high levels required to recruit A-beta nociceptors. To extend the preliminary findings above, a multicenter, double-blind, randomized controlled trial in the United States has just been completed, and results are forthcoming (ClinicalTrials.gov ID #NCT02924129). A salient factor in closed-loop SCS may be that its sensing is dependent on activation of fibers that generate perceptible paresthesias; the applicability of closed-loop feedback technology to paresthesia-free SCS waveforms, then, remains to be seen.

## System Miniaturization

**Figure 1:**
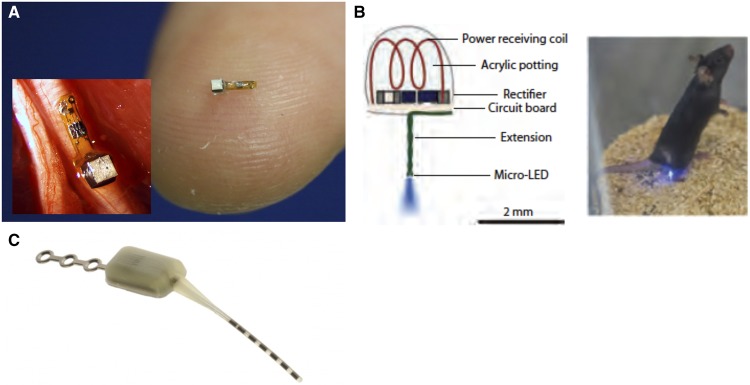
Forward looking approaches to neuromodulation. A) A “neural dust” miniaturization of systems that can be externally powered for various recording and, potentially, stimulating applications [96]. B) An optogenetic approach to modulate specific cells in the central nervous system to induce analgesia. Image used with permission from Montgomery et al. [97]. C) All-in-one small stimulator designed specifically for sphenopalatine ganglion stimulation [98].

Modern neurostimulators consist of leads and an implanted pulse generator, which have evolved from radiofrequency-powered devices to internal battery systems that are both primary cell and rechargeable. Some contemporary technology is now shifting back to externally powered devices for peripheral nerve stimulation and for other neurostimulation applications or patients in whom internal batteries would be anatomically challenging or unwanted or perceived as unsightly by patients. Implantable nerve stimulators have been the first-generation miniature wireless stimulators to reach patients for peripheral applications such as chronic hemiplegic shoulder pain and central applications such as sphenopalatine ganglion stimulation for cluster headache (Figure 1) [99–103]. These externally powered devices have been developed with a wide range of delivery options, from percutaneous ultrasound-guided options to open implants. In peripheral stimulation, there are significant advantages to externally powered devices, and the versatility afforded by externalizing certain components has significant implications in miniaturization. The data supporting the clinical efficacy of these devices are beyond the scope of this paper, but the concepts of versatility and upgradeability embodied by their existence are paramount to the advanced concepts described below, including injectable neurostimulators, microelectrode arrays, and optogenetics.

**Figure 2:**
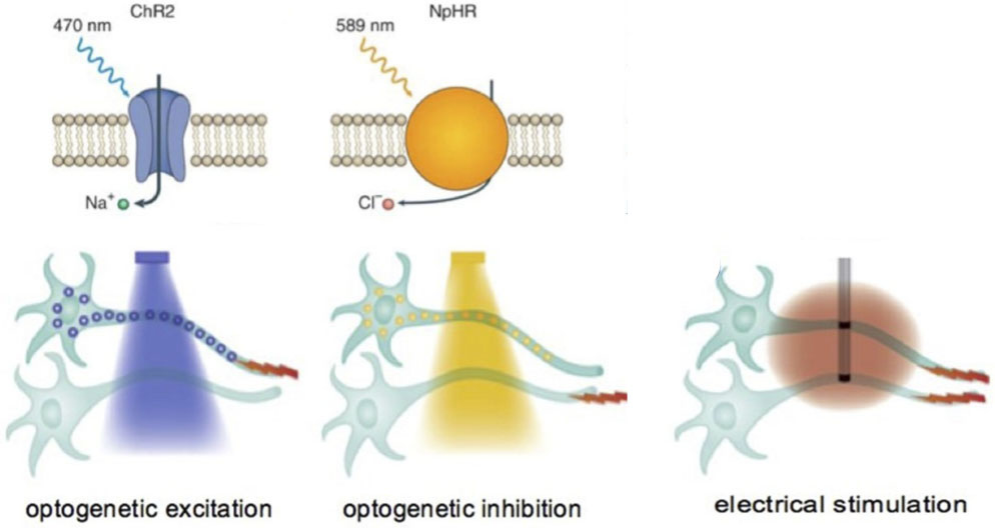
Optogenetic approaches to modulating neural function. Light-sensitive ion channels (channelrhodopsins) can be expressed in neurons of a specific phenotype that, in turn, allow cell membranes to become depolarized (excited) or hyperpolarized (inhibited) by applying light to the cell. Specific wavelengths of light (blue, yellow, etc.) can be used to excite or inhibit cells based upon the type of channelrhodopsin expressed in the neuron [175].

Despite their relatively compact sizes, the above neurostimulation systems are still orders of magnitude larger than cells. There have been significant advances in materials that allow for size scales of devices to approach the dimensions of cells, which may improve the selectivity of stimulation [104]. Microelectrodes with arrays of varying length have been created to target different fascicles, perhaps ideal for peripheral nerve stimulation applications. Current microelectrode arrays (0.5–5 mm in length and 15–50 µm wide) are capable of neural recording and stimulation, but they have been observed in vivo to trigger a limiting foreign body response in brain tissue that is similar to neurotrauma and neurodegeneration [104]. This response can be mitigated by decreasing the size and enhancing the flexibility of the implant; however, this presents challenges for device fabrication, device delivery, ability to handle appropriate stimulation current levels, and long-term stability. Furthermore, there are challenges with accurate positioning and tracking of the devices over time in addition to programming.

Conceptually, an injectable neurostimulator requires most, if not all, of the components of its full-sized progenitors to be contained in a package small enough to be delivered percutaneously. This includes two or more electrodes, a telemetry module, sensors, a power supply, a microprocessor, a data transceiver, and power management/recharging capabilities [105]. Recently, a working ultramicro neural sensor was developed [106,107] with a microelectronics platform that utilizes ultrasound wavelengths to both power and communicate with the device (Figure 1). The entire footprint of the unit was designed for nerve recordings to fit onto a single nerve. The so-called “neural dust” embodies the rapidly decreasing scale of neurorecording and neurostimulation platforms. As these become nanoscale, the potential for injectable distribution becomes more of a reality, as does the discrete targeting of specific cell groups.

## Optogenetics

All the neuromodulation techniques that have been described thus far involve the use of electricity for neural manipulation. In 2005, Ed Boyden and Karl Deisseroth reported a technique by which neurons not previously sensitive to light could be genetically manipulated to express light-sensitive ion channels and, thus, become depolarized by photons [108]. The field of optogenetics has exploded with the utility of this technique, and it is currently being utilized for both basic discovery within systems neuroscience and as a part of therapy development efforts. Conceptually, virally mediated transfection of genes coding for light-sensitive proteins (opsins, similar to those in the eye) conjugated to specific ion channels is achieved in neurons via promotor-driven expression. Thus, very targeted expression can be achieved within a specific neuronal phenotype, even if those cells are interspersed heterogeneously within a group of other cells. The capacity to specifically generate very targeted expression results in the ability to use light to modulate activity in a very select population of neurons (Figure 2). This approach has been utilized to demonstrate the feasibility of selectively manipulating neurons controlling nociception and pain within the peripheral and central nervous system [97,109–113]. Pain can be both induced or inhibited based upon the cells that are targeted and the specific channel-rhodopsins that are expressed. Although it is still early, the approach of using light to specifically inhibit or activate neurons provides a powerful tool to control neural function and induce analgesia. Beyond the design and construction of viral vectors that can selectively induce the expression of light-sensitive channels and convey optical sensitivity to neurons, there is also a significant effort to miniaturize light sources on the device side of this therapeutic approach in order to make the application anatomically flexible and battery-free. Overall, this technique still has developmental hurdles to overcome, and it is yet to be seen if the ability to target specific neurons in discrete anatomical regions will translate into effective pain management in humans.

## Neural Targets

### Dorsal Root Ganglion

The dorsal root ganglion has emerged as an important neuromodulation target [5,114–116]. The combination of special anatomy and physiology of the primary sensory neuron at the level of the ganglion demonstrates the multiple aspects to stimulation that need to be tailored and accounted for when designing a stimulation therapy. There are several papers that address the clinical and mechanistic aspects of DRG stimulation in this special edition. As such, this neural target will not be fully addressed here.

### Vagal Nerve Stimulation—Immune Function and Headache

Cytokine production by the immune system contributes to both health and disease. The nervous system, via an inflammatory reflex of the vagus nerve, can inhibit cytokine release and thereby prevent tissue injury and death. The efferent neural signaling pathway is termed the cholinergic anti-inflammatory pathway. Stimulation of the vagus nerve prevents the damaging effects of cytokine release in animal models of sepsis, endotoxemia, ischemia/reperfusion injury, hemorrhagic shock, arthritis, and other inflammatory syndromes [117].

Using this information, and the fact that it previously was unknown whether directly stimulating the inflammatory reflex in humans inhibits tumor necrosis factor (TNF)–α production, Koopman et al. demonstrated that peripheral blood production of TNF-α, interleukin (IL)-1β, and IL-6 was inhibited in epilepsy patients with an implantable left cervical vagus nerve–stimulating device [118]. Thereafter, it was shown that vagus nerve stimulation in rheumatoid arthritis patients significantly inhibited TNF-α production for up to three months and disease severity improved significantly, establishing that the inflammatory reflex modulates TNF-α production and modulating the vagus nerve reduces inflammation in humans. These findings suggest the possibility of using mechanism-based neurostimulation devices in the treatment of rheumatoid arthritis (RA) and potentially other autoimmune and auto-inflammatory diseases such as inflammatory bowel disease. In fact, there is currently a US pilot trial (Safety and Efficacy of Vagus Nerve Stimulator in Patients with Rheumatoid Arthritis) evaluating vagus nerve stimulation (VNS) in the treatment of RA (ClinicalTrials.gov NCT03437473). Mechanistically, VNS activates the cholinergic anti-inflammatory pathway that ameliorates cytokine-mediated disease and is dependent on an intact splenic nerve [119]. Therefore, the splenic nerve may be another target for neuromodulation in the treatment of autoimmune inflammatory diseases underlying chronic pain conditions.

The vagus nerve is also a target for the treatment of headache disorders. The idea of stimulating the vagus nerve to treat headache first came from the unanticipated observation that patients being treated with a vagus nerve stimulator for epilepsy had reduced severity and incidence of their coexistent migraines while using the stimulator [120]. Furthermore, noninvasive vagus nerve stimulation (nVNS) at the carotid artery can be used for prophylactic and abortive treatment of cluster and migraine headaches with reduced cortical spreading depression and demonstrated progression in efficacy over time [121].

Speculations on the pathophysiological mechanisms that mediate the effect of nVNS in migraine mainly originate from animal studies. The main mechanistic hypothesis is that the afferent anatomical connection between the vagus nerve and the trigeminal nucleus caudalis [122] and the nociceptive inputs from the dura mater terminating in the nucleus tractus solitarius [123] are interrupted. Neurophysiological evidence showing a reduction in glutamate levels and neuronal firing in the spinal trigeminal nucleus secondary to continuous vagus stimulation [124] could justify vagal ascending antinociceptive effects. This seems to be further confirmed by studies in rats, which have demonstrated that vagus nerve stimulation can reduce pain [125] and allodynia [126] in the trigeminal area.

### SPG Stimulation

The sphenopalatine ganglion (SPG) is another neuromodulatory target for the treatment of headaches. The SPG has been targeted with injections of local anesthetic [127] and has been subject to neurolysis/radiofrequency ablation [128] to abort and reduce migraine and cluster headaches. Recent sham-controlled RCTs have shown significant ability to provide pain relief and reduce the number of headache days when utilizing neurostimulation of the SPG [100,129]. These results have been maintained for up to two years [129,130]. Tepper et al. performed a pilot study to evaluate the effectiveness of SPG stimulation in the treatment of migraine headaches. Migraine attacks were induced in 11 patients with refractory headache. They were then treated with electrical stimulation through a needle introduced into the sphenopalatine fossa via an infrazygomatic approach. The treatment was effective in less than half the patients, with two reporting a completely aborted attack and three with significant pain relief. These results might have been caused by suboptimal lead placement, inadequate lead design for the pterygopalatine fossa, and inclusion of medication overuse in a subgroup of patients [131]. Given the role that the SPG plays in autonomic regulation within the head (parasympathetic outflow to the periorbital, nasal, meningeal, and cerebral blood vessels) [132–134], the SPG is an intriguing target that may benefit from advances in neuromodulation, including application of different waveforms.

### Trigeminal System Stimulation

It is reasonable to assume that neuromodulation for the treatment of trigeminal neuralgia and intractable facial pain might provide good relief, as neuroablative procedures of the trigeminal nucleus caudalis and spinal trigeminal tract have resulted in pain relief [135–139]. Despite this assumption, the body of evidence in the literature for Trigemino-cervical complex (TCC) region stimulation for facial pain is relatively small [136].

Upper cervical SCS is one approach that has been attempted to modulate the TCC. A retrospective case series found that 75% of implanted patients continued to derive benefit for up to 10 years after implantation [140]. Velasquez et al. conducted a retrospective, consecutive, single-center series of 12 patients with trigeminal neuropathy treated with upper cervical spinal cord stimulation [141]. The average coverage in the pain zone was 72%, and the median baseline, trial, and postoperative numeric rating scale (NRS) scores were 7, 3, and 3, respectively. The mean reduction in the numeric rating scale from baseline was 4 points, resulting in an average 57.1% pain reduction. The long-term treatment failure rate was 25%. These results are encouraging and point to another approach to modulating the trigeminal system in the treatment of cranial pain conditions. Recently, noninvasive methods have been utilized to stimulate the supraorbital nerve, a branch of V_1_, that eventually feeds into the trigeminal system. In an open label study, Chou et al. demonstrated the safety and efficacy of an external device to stimulate the supraorbital nerve to treat acute migraine headaches [142]. Similar findings were also observed in a blinded, pilot RCT [143]. Subsequent research has demonstrated alterations in brain metabolism, with this type of noninvasive neuromodulation providing further support of the physiologic mechanisms underlying the approach [144].

### Neuromodulation for Joint Pain and Preventing Articular Disease Progression

Pulsed radiofrequency (PRF) to address pain has been applied to multiple anatomies, including intraspinal and extraspinal articular structures, the shoulder, and the knee [145–147]. Several studies have shown that PRF of the suprascapular nerve may relieve shoulder pain and can improve mobility of the shoulder joint [102,146,148]. Intra-articular PRF has resulted in significant and durable pain relief in a majority of patients with joint pain [147]. Although the exact mechanism is unclear, it may be related to the exposure of immune cells to low-strength RF fields, stimulating an anti-inflammatory effect.

With the knowledge of pulsed radiofrequency of neural structures (including those innervating joints), it seems reasonable to apply current and future implantable continuous neuromodulation devices to treat joint pain with intra-articular implantation. Apart from providing palliative treatment effects such as pain relief, it is also possible that stimulation may help treat underlying inflammatory mechanisms in joints [149]. With miniaturization or the advent of thin film-like devices, intra-articular implantation may be possible. Joints are generally anatomies with a high degree of movement. Thus it is important to consider whether the joint itself, or the nerve branches innervating the structure, should be the location of the neuromodulation.

### Occipital Nerve Stimulation

The greater and lesser occipital nerves send afferent projections through the C2-3 dorsal roots, which then make synaptic connections in the upper cervical spinal cord, including cells in the TCC [150,151]. Stimulation of this afferent pathway, in turn, may lead to an ability to modulate TCC function and downstream trigeminal dysfunction in head and facial pain. Occipital nerve stimulation (ONS) has been extensively used to treat various headaches, including cervicogenic, migraine, cluster, and occipital neuralgia, since the initial report by Weiner and Reed [152]. A recent systematic review with meta-analysis showed that there is favorable but low-quality evidence to support use of tonic ONS for decreasing the intensity and frequency of headache pain associated with intractable primary headaches [153]. Another systematic review makes a level 3 recommendation for the use of tonic ONS as a treatment option for medically refractory occipital nerve pain [154]. Puledda and Goadsby reviewed the current evidence and status for neuromodulation devices for the acute and preventive treatment of migraine. They concluded that more studies with appropriate blinding strategies are needed to confirm the results of new treatments [155]. Multiple attempts have been made to demonstrate the efficacy of occipital nerve stimulation through sham-controlled studies, yet these have been met with failure to meet the primary end point [156,157]. Likely reasons for these failures include poor patient selection and inclusion, primary outcome measures, method of sham control, and variability in the outcomes that may have been better controlled. Interestingly, one study examined the effects of a burst stimulation pattern during ONS in healthy volunteers and found that there were different patterns of cerebral activation, suggesting that, like SCS, burst may provide a differential clinical outcome [158]. In general, however, further work needs to be completed on novel stimulation paradigms in ONS.

### Application of Novel Waveforms to Peripheral Targets

As previously discussed, newer stimulation patterns of the dorsal column have led to improved pain relief and function over classic lower-frequency tonic stimulation. Physiologically, a particular structure may require a specific stimulation pattern and stimulation timing that are different from another target. For example, as opposed to spinal cord stimulation, VNS may be used for short sessions periodically throughout the day (although the optimal treatment regime has not yet been identified). Thus, because of the neural target and the desired effect, a specific, and minimal, stimulation paradigm can be employed. Applying ideal target-specific stimulation patterns may improve outcomes in areas where current neuromodulation has shown less than impressive results. Another example of this comes from Kent et al., who applied computational modeling to elucidate the mechanisms of action of DRG stimulation for low- and high-frequency waveforms. Afferent signals measured in nociceptive neurons could be suppressed during delivery of 40-Hz LFS, as well as 1-kHz and 10-kHz HFS, suggesting that high-frequency DRG stimulation may not provide any more relief in chronic pain conditions [159]. On the other hand, high-frequency stimulation of peripheral nerves causes a complete conduction block [160] and relieves postamputation pain [161]. Applying different waveforms or stimulation patterns may improve outcomes in painful conditions underserved by present neurostimulation targeting. Revisiting these targets with new patterns may improve outcomes. Thus, as new neural targets are identified, it will be incumbent to understand not only the best physical embodiment of the neuromodulation device for the intended target, but also the most effective stimulation pattern and the timing of the pattern to other temporally important physiological biomarkers (e.g., hormonal fluctuations, immunological rhythms, patterned neural events).

## Biomarkers and Neuromodulation for Pain

Chronic neuropathic pain treatment has had limited success, due in part to a relatively poor understanding of the mechanisms underlying the development and maintenance of the condition. Additionally, the effectiveness of neuropathic pain management regimens and procedures can be difficult to determine, due to the subjective nature of pain perception and a lack of standardized assessment tools. Study in the fields of neuroimaging, genomics, and molecular biology is helping identify unique biomarkers and signatures that could lead to improved objectivity and standardization in measuring nociceptive correlates. Surprisingly, relatively little research has been conducted on the impact of SCS on various objective biomarkers in the form of easily obtainable and measurable physiologic responses. Most recently, there have been studies beginning to examine the impact of SCS on various protein levels in cerebrospinal fluid [162–165], circulating and regional inflammatory intermediates [166,167], and gene transcripts [168,169]. Regional changes in cerebral activation patterns have also been examined [46,170–172], as well as electrophysiological analyses in humans [45,91,95,173,174]. Some of this work has been used to examine neuromonitoring for safety during implantation. Beyond the large body of mechanistic work that is outside the scope of this review, it is unclear how biomarkers might play a role in spinal cord therapy stimulation. Some possibilities include markers to help determine stimulation candidates or predict therapy success, provide an objective measure of a physiologic component or proxy of pain or an indicator to help determine optimal therapy delivery. As this aspect of neuromodulation continues to develop, it will be interesting to note how these types of biomarkers may play an increasing role in the therapy.

## Conclusions

Technological advancements in microelectronics, feedback-based system design, biomimetic stimulation patterns, and the interplay between genomics and device-based therapies are transforming how neuromodulation is being conceived. Miniaturization and noninvasive therapies are providing the templates for increasingly smaller devices or noninvasive therapies that do not require surgery. As our understanding of how the interplay between neural targets and stimulation patterns continues to develop, the landscape of the therapeutic arsenal that physicians have at their disposal will most certainly continue to grow.

As technology and approaches to neuromodulation advance, it is clear that systems need to be more flexible in their ability to produce multiple programmable outputs in order to not only tailor treatment to specific diagnoses and conditions, but also individual patients. The ability to shift stimulation output from one pattern to the next could also potentially help avoid therapeutic fatigue, which is the primary reason for device explants. To most effectively do this, we echo comments from De Ridder in a call for software-driven systems, as opposed to hardware being the limiting factor [82]. Having hardware platforms capable of delivering multiple evidence-based therapeutic modalities will be a benefit to patients. In concert with this, various biomarkers may become an increasingly important component of the treatment algorithm to help understand how to individualize neuromodulation therapy. This includes understanding why therapies are failing over time. As such, the need for continued high-quality clinical evidence will also be required to establish the right foundation for this growing field. This will also include, as appropriate and possible, the use of sham controls in clinical studies. Lastly, we continue the call to better understand the physiologic underpinnings of these therapies through basic research to help understand how to continually improve upon existing technologies and drive discovery in areas not yet explored. To this end, it remains to be seen if neuromodulation therapies can positively prevent or impact underlying disease mechanisms and move from a palliative treatment paradigm to a disease prevention model.

## Notes

Funding sources: This manuscript was prepared with financial support from Abbott.

Conflicts of interest: Dr. Fishman is a consultant for Abbott, Biotronik, Cornerloc, Medtronic, and Nevro; funded research for Abbott, Biotronik, Medtronic, Nuvectra, Stimgenics; majority stock holder of Celéri Health, Inc. and a minority stock holder of Thermaquil. Dr. Antony is a consultant and principle investigator for Abbott; is an advisory board member for Boston Scientific. Dr. Esposito is a consultant for Abbott and Flowonix. Dr. Deer is a minority stock holder in Axonics, Bioness, Nalu, Saluda, Spinethera, Vertos, Vertiflex, Cornerloc; is a consultant for Abbott, Saluda, Flowonix, Vertos, and Vertiflex; has received funded research from Abbott, Mainstay, Vertiflex, Vertos, Saluda. Dr. Levy is a minority stock holder in Bioness, Nalu, Saluda, Vertos; a consultant for Abbott, Mainstay, Nalu, Nuvectra, Saluda, Vertos.

Supplement sponsorship: This article appears as part of the supplement “Neuromodulation of the Spine and Nervous System” sponsored by Abbott.
